# Adverse Prehospital Events and Outcomes After Traumatic Brain Injury

**DOI:** 10.1001/jamanetworkopen.2024.57506

**Published:** 2025-01-31

**Authors:** Amelia W. Maiga, Hsing-Hua Sylvia Lin, Stephen R. Wisniewski, Joshua B. Brown, Ernest E. Moore, Martin A. Schreiber, Bellal Joseph, Chad T. Wilson, Bryan A. Cotton, Daniel G. Ostermayer, Brian G. Harbrecht, Mayur B. Patel, Jason L. Sperry, Francis X. Guyette, Henry E. Wang

**Affiliations:** 1 Division of Acute Care Surgery, Department of Surgery, Section of Surgical Sciences, Vanderbilt University Medical Center, Nashville, Tennessee; 2 Critical Illness, Brain Dysfunction, and Survivorship Center, Vanderbilt Center for Health Services Research, Vanderbilt Institute for Medicine and Public Health, Vanderbilt University Medical Center, Nashville, Tennessee; 3 Department of Anesthesiology and Perioperative Medicine, University of Pittsburgh, Pittsburgh, Pennsylvania; 4 Department of Epidemiology, School of Public Health, University of Pittsburgh, Pittsburgh, Pennsylvania; 5 Department of Surgery, University of Pittsburgh, Pittsburgh, Pennsylvania; 6 Department of Surgery, Ernest E. Moore Shock Trauma Center at Denver Health, Denver, Colorado; 7 Division of Trauma, Critical Care, and Acute Care Surgery, Oregon Health & Science University, Portland; 8 Division of Trauma, Surgical Critical Care, Burns, and Acute Care Surgery, Department of Surgery, University of Arizona, Tucson; 9 Department of Surgery, Baylor College of Medicine, Houston, Texas; 10 Division of Acute Care Surgery and Center for Translational Injury Research, Department of Surgery, McGovern Medical School at the University of Texas Health Science Center, Houston; 11 Department of Emergency Medicine, McGovern Medical School at the University of Texas Health Science Center, Houston; 12 Department of Surgery, University of Louisville, Louisville, Kentucky; 13 Department of Emergency Medicine, University of Pittsburgh, Pittsburgh, Pennsylvania; 14 Department of Emergency Medicine, The Ohio State University, Columbus

## Abstract

**Importance:**

While national guidelines recommend avoidance of hypoxia, hypotension, and hypocarbia in the prehospital care of traumatic brain injury (TBI), limited data validate the association of these adverse physiologic events with TBI outcomes.

**Objective:**

To validate the associations of prehospital hypoxia, hypotension, and hypocarbia with TBI outcomes in a US national trauma network.

**Design, Setting, and Participants:**

This cohort study examined data from 8 level I trauma centers and their affiliated ground and air emergency medical services (EMS) agencies in the Linking Investigations in Trauma and Emergency Services (LITES) Network from January 1, 2017, to June 30, 2021. Adult patients (aged ≥18 years) with confirmed TBI (head Abbreviated Injury Score [AIS] of 1-6) and Injury Severity Score (ISS) of at least 9 were included. Interfacility transfers and patients who underwent prehospital cardiopulmonary resuscitation were excluded. Data were analyzed between April 20, 2022, and November 27, 2023.

**Exposures:**

Adverse prehospital TBI events, including hypoxia, hypotension, or hypocarbia.

**Main Outcomes and Measures:**

The primary outcomes were death in the emergency department (ED), hospital death, and unfavorable discharge disposition. Log-binomial regression models were used to estimate the association between adverse TBI events and outcomes, adjusting for sex, race and ethnicity, age, study site, transport mode, initial Glasgow Coma Scale, ISS, head AIS score, injury mechanism, and multiple trauma.

**Results:**

The analytic cohort included 14 994 patients (median [IQR] age, 47 [31-64] years; 71% male; median [IQR] head AIS, 3 [2-4]). Patients with adverse TBI events included 12% (1577 of 13 604) with hypoxia, 10% (1426 of 14 842) with hypotension, and 61% (650 of 1068) with hypocarbia among those with advanced airway management. Patient outcomes included 2% (259 of 14 939) who died in the ED, 12% (1764 of 14 623) who died in the hospital, and 25% (3705 of 14 623) with an unfavorable discharge disposition. Hypoxia (adjusted relative risk [ARR], 2.24; 95% CI, 1.69-2.97), hypotension (ARR, 2.05; 95% CI, 1.54-2.72), and hypocarbia (ARR, 7.99; 95% CI, 2.47-25.85) were associated with increased risks of ED death. Each adverse TBI event exposure was also associated with higher risks of hospital death and unfavorable discharge disposition.

**Conclusions and Relevance:**

In this multicenter cohort study, prehospital hypoxia, hypotension, and hypocarbia were associated with poorer TBI outcomes. These results underscore the importance of optimal oxygenation, ventilation, and perfusion in prehospital TBI care.

## Key Points

**Question:**

Are prehospital hypoxia, hypotension, and hypocarbia associated with poor outcomes after traumatic brain injury (TBI)?

**Findings:**

In this cohort study of 14 994 adults with confirmed TBI, prehospital hypoxia, hypotension, and hypocarbia were each associated with increased risks of death in the emergency department, hospital death, and unfavorable discharge disposition.

**Meaning:**

These findings suggest that optimal oxygenation, ventilation, and perfusion are crucial to mitigating secondary injury in prehospital TBI care.

## Teaser

This cohort study examines the association of prehospital hypoxia, hypotension, and hypocarbia with traumatic brain injury outcomes in US adult patients.

## Introduction

Traumatic brain injury (TBI) is a major international public health problem, affecting nearly 70 million people annually.^1,2,3,4^ Severe TBI may cause death or irreversible disability within the first few hours after injury.^5,6^ Patients with TBI commonly receive initial care from emergency medical services (EMS). The Brain Trauma Foundation (BTF) has developed evidence-based guidelines for the prehospital treatment of patients with suspected TBI that are designed specifically to prevent secondary brain injury and avoid secondary iatrogenic insults to the brain.^5,7^

It is crucial to anticipate, prevent, and rapidly reverse hypoxia (<90% arterial oxygen saturation [Sao_2_]), hypotension (<90 mm Hg systolic blood pressure [SBP]), and hyperventilation (manifesting as hypocarbia [end-tidal CO_2_ <35 mm Hg]) in any patient with suspected TBI.^5^ Despite widespread dissemination of the BTF guidelines, patients with TBI commonly experience prehospital hypoxia, hypocarbia, and/or hypotension.^8^ The Excellence in Prehospital Injury Care (EPIC) Study, which tested the implementation of many of the principles of the BTF guidelines, supported the value of avoiding hypoxia, hypotension, and hypocarbia in prehospital TBI care.^9^

The multicenter Linking Investigations in Trauma and Emergency Services (LITES) Network is a contemporary consortium of trauma systems and their affiliated ground and air EMS systems. The aim of this study was to investigate the association of prehospital hypoxia, hypotension, and hypocarbia with TBI outcomes in the LITES Network.

## Methods

### Study Design, Setting, and Data Source

This multicenter cohort study was deemed exempt by the University of Pittsburgh Institutional Review Board, including exemption from informed consent due to the use of deidentified observational data. We followed the Strengthening the Reporting of Observational Studies in Epidemiology (STROBE) reporting guideline.^10^

We used data from the LITES Task Order One (TO1) Network. Supported by the US Department of Defense, LITES TO1 is a collaborative research network of 8 level I trauma centers and more than 30 affiliated ground and air EMS agencies. Participating trauma centers of the network are located in Pittsburgh, Pennsylvania (University of Pittsburgh Medical Center); Denver, Colorado (Denver Health); Portland, Oregon (Oregon Health & Science University); Tucson, Arizona (University of Arizona); Louisville, Kentucky (University of Louisville); Houston, Texas (Memorial Hermann-Texas Medical Center and Baylor College of Medicine); and Nashville, Tennessee (Vanderbilt University Medical Center).

The LITES TO1 registry is a standardized prehospital dataset of patients treated at each trauma center. The registry includes patients aged 18 to 90 years with an Injury Severity Score (ISS) greater than 9 (indicating moderate to severe injury) transported by and receiving care from affiliated EMS agencies. The LITES TO1 registry excluded women known to be pregnant and incarcerated individuals. Prehospital data originated from National EMS Information System–compliant electronic health records. Inpatient data originated from the American College of Surgeons Trauma Quality Improvement Program (TQIP), a national multi-institutional database of all patients with trauma collected from participating trauma centers. At each participating trauma center, trained data registrars compiled patient- and center-related variables quarterly over a period of 5 years from 2017 to 2021. The Epidemiology Data Center at the University of Pittsburgh linked prehospital and inpatient data via probabilistic methods using demographic and injury information.

### Study Population

For this study, we included adult patients with TBI who were transported directly to a trauma center by ground or air EMS. We defined TBI as head Abbreviated Injury Score (AIS) of 1 to 6, which is the anatomic component of the ISS that corresponds to head injuries. Head AIS scores were determined by expert trauma registrars as part of each participating hospital’s TQIP database. We excluded patients not transported by EMS or transferred from other hospitals. We also excluded patients who received prehospital cardiopulmonary resuscitation and those missing prehospital vital signs.

### Exposures

Adverse prehospital events based on BTF guidelines, including hypoxia, hypotension, and/or hypocarbia, were the primary exposures. Hypoxia included any prehospital Sao_2_ less than 90%. Hypotension included any prehospital SBP less than 90 mm Hg. Hypocarbia included any end-tidal CO_2_ level less than 35 mm Hg after intubation or supraglottic airway insertion and during the entire remaining prehospital phase of care. Hypocarbia was only assessed in the subset of the population undergoing prehospital advanced airway management. We defined adverse TBI care events as the occurrence of hypoxia, hypotension, or hypocarbia.

### Study Outcomes

The TBI outcomes included (1) death in the emergency department (ED) (ie, prior to hospital admission), (2) hospital death, and (3) unfavorable discharge disposition. Favorable discharge disposition included hospital discharge to short-term inpatient care, an intermediate care facility, inpatient rehabilitation, or home or self-care (routine discharge, including discharge to law enforcement or psychiatric care). Unfavorable discharge disposition included death or admission to a skilled nursing facility, hospice care, or a long-term-care hospital.^11,12^

### Statistical Analysis

We summarized continuous variables as medians with interquartile ranges and categorical variables using frequencies and percentages. We used the Kruskal-Wallis test or Pearson χ^2^ test to examine whether distributions of continuous or categorical variables, respectively, differed by prehospital TBI adverse care events (yes vs no). Using log-binomial regression models, we estimated the relative risks (RRs) and 95% CIs for the associations between each independent adverse prehospital care exposure (ie, any adverse care event, hypoxia, hypotension, hypocarbia) and each TBI outcome (ie, death in the ED, death in the hospital, unfavorable discharge disposition). Confounders adjusted for in each model included age, sex, race and ethnicity, transport mode (ground vs air), initial Glasgow Coma Scale (GCS) score, ISS, trauma mechanism (blunt vs penetrating), multiple trauma (TBI plus injury to at least 1 other body region with an AIS ≥3), and the 8 study sites. Race and ethnicity (American Indian, Asian, Black or African American, Native Hawaiian or Other Pacific Islander, White, or other) were extracted directly from the TQIP database and were self-reported or identified by a family member. The categories of race were based on the 2010 US Census Bureau definitions.^13^ Race and ethnicity were included as variables as they are sometimes associated with trauma-specific outcomes. Because the outcomes of different head injuries vary, we stratified the analyses by head AIS: moderate to severe (AIS 1-4) and critical (AIS 5-6). Due to the limited number of events, we combined AIS 1-2 and 3-4 for the stratification.

Log-binomial regression models are commonly used to estimate relative risk (risk ratios) in binary outcomes, especially when studying common events where the odds ratio from logistic regression may overestimate the effect size.^14^ Assumptions of log-binomial regression models (ie, independence of observations, linear relationship between the predictors and the log of the probability of the outcome [log risk], estimated probabilities within the 0-1 range) were assessed, and all were appropriate for the analyses in this study. Missingness was less than 5% for all analytic variables except initial field GCS (10.6%) and hypoxia (9.3%), and data may have been missing at random. Complete case analysis is appropriate when the level of missingness is low (ie, <20%), particularly in large datasets where low missingness has a minimal impact on bias and maintains good precision.^15^ All analyses were conducted between April 20, 2022, and November 27, 2023, using SAS, version 9.4 (SAS Institute Inc). A 2-sided *P* < .05 was considered statistically significant.

## Results

### Study Population

Of the 77 596 patients in the LITES TO1 cohort, there were 35 191 adults with TBI. We excluded 1112 not transported by EMS, 14 158 not transported from the scene, 1019 who received prehospital cardiopulmonary resuscitation, and 3908 with missing prehospital vital signs. The final analytic cohort included 14 994 individuals from the TQIP database (Figure) and 47 549 prehospital vital records from EMS agencies.

**Figure.**
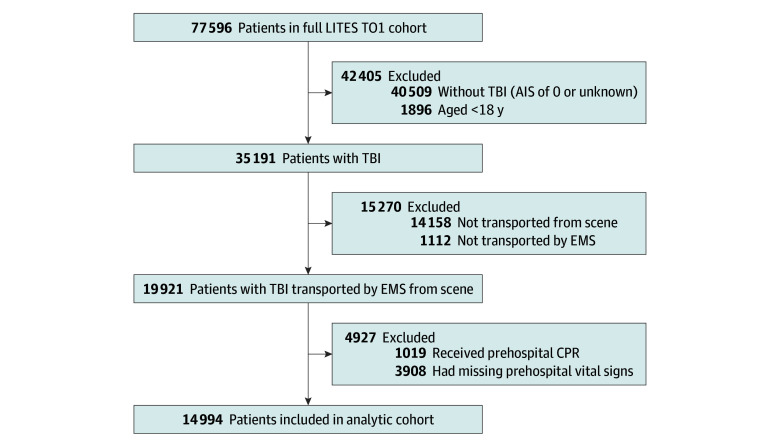
Study Flowchart for the Analytic Cohort AIS indicates Abbreviated Injury Score; CPR, cardiopulmonary resuscitation; EMS, emergency medical services; LITES TO1, Linking Investigations in Trauma and Emergency Services Task Order One; TBI, traumatic brain injury.

### Demographics

Patient demographics and injury characteristics are shown in Table 1. The overall population was 29% female and 71% male (median age, 47 years; IQR, 31-64 years), and 1% identified as American Indian, 2% as Asian, 15% as Black or African American, 71% as White, and 12% as other race and ethnicity. The median of initial field GCS was 14 (IQR, 11-15); ISS was 17 (IQR, 12-26), with 4401 patients (29%) having an ISS of at least 25; and head AIS was 3 (IQR, 2-4). A total of 3946 patients (26%) were transported by air EMS. Patients experiencing adverse prehospital hypoxia, hypotension, or hypocarbia compared with those who did not had a significantly lower median GCS (9 [IQR, 3-14] vs 15 [IQR, 13-15]; *P* < .001), higher median ISS (25 [IQR, 3-14] vs 17 [IQR, 11-24]; *P* < .001), higher proportion of multiple-trauma TBI (1874 [65%] vs 5794 [48%]; *P* < .001), and higher proportion of penetrating (350 [12%] vs 606 [5%]; *P* < .001) vs blunt injuries (2480 [87%] vs 11 179 [93%]; *P* < .001). These patients with adverse prehospital exposures were also more likely to be transported by helicopter vs those without adverse prehospital exposures (1316 [46%] vs 2630 [22%]; *P* < .001).

**Table 1.** Demographics and Injury Characteristics of Patients With Acute TBI, Stratified by the Occurrence of a Prehospital Adverse Care Event

Variable	No. of patients (%)		
	All (N = 14 994)	Any prehospital adverse care event (hypoxia, hypotension, and/or hypocarbia)	
		No (n = 12 114)	Yes (n = 2880)
Sex			
Female	4354 (29)	3551 (29)	803 (28)
Male	10 640 (71)	8563 (71)	2077 (72)
Age, median (IQR), y	47 (31-64)	48 (31-65)	45 (30-62)
Race and ethnicity^a^			
American Indian	105 (1)	85 (1)	20 (1)
Asian	231 (2)	201 (2)	30 (1)
Black or African American	2213 (15)	1830 (15)	383 (14)
Native Hawaiian or Other Pacific Islander	15 (<1)	10 (<1)	5 (<1)
White	10 378 (71)	8371 (70)	2007 (72)
Other^b^	1777 (12)	1418 (12)	359 (13)
Transport mode			
Ground ambulance	11 048 (74)	9484 (78)	1564 (54)
Helicopter ambulance	3946 (26)	2630 (22)	1316 (46)
Initial field GCS,^a^^,^^c^ median (IQR)	14 (11-15)	15 (13-15)	9 (3-14)
ISS,^a^^,^^d^ median (IQR)	17 (12-26)	17 (11-24)	25 (17-34)
Head AIS,^e^ median (IQR)	3 (2-4)	3 (2-4)	3 (2-4)
Injury type^a^			
Blunt	13 659 (92)	11 179 (93)	2480 (87)
Penetrating	956 (6)	606 (5)	350 (12)
Burn or other or unspecified	231 (2)	194 (2)	37 (1)
Body region			
Isolated TBI	7326 (49)	6320 (52)	1006 (35)
Multisystem injury: TBI and other body AIS ≥3	7668 (51)	5794 (48)	1874 (65)

Abbreviations: AIS, Abbreviated Injury Scale; GCS, Glasgow Coma Scale; ISS, Injury Severity Score; TBI, traumatic brain injury.

^a^ Missingness for race and ethnicity, 275 (1.8%); for GCS, 1595 (10.6%); and for injury type, 148 (1.0%).

^b^ Cannot be further specified.

^c^ Scale of 3 to 15, with lower scores indicating more severe brain injury.

^d^ Scale of 3 to 75, with higher scores indicating more severe multiple trauma.

^e^ Scale of 1 to 6, with a score of 1 or 2 indicating mild injury, a score of 3 or 4 indicating moderate injury, and a score of 5 or 6 indicating severe injury.

### Adverse Prehospital TBI Care Events

Table 2 shows the prevalence and overlap of comorbid prehospital hypoxia, hypotension, and hypocarbia among adults with TBI in the LITES TO1 registry. Overall, 12% (1577 of 13 604) experienced prehospital hypoxia (Table 2), with a median lowest prehospital Sao_2_ of 82% (IQR, 75%-87%) and a median of 3 prehospital Sao_2_ measurements (IQR, 1-6) (eTable 1 in Supplement 1). Patients without hypoxia had a median minimum prehospital Sao_2_ of 96% (IQR, 95%-99%). Prehospital hypotension was experienced by 10% of patients with TBI (1426 of 14 842), with a median lowest prehospital SBP of 75 mm Hg (IQR, 63-81 mm Hg) and a median of 4 (IQR, 1-7) prehospital SBP readings recorded. Among 14 994 patients, 83% did not have prehospital hypoxia or hypotension, 8% experienced hypoxia only, 7% experienced hypotension only, and 3% had both hypoxia and hypotension.

**Table 2.** Prevalence of Each and Combined Prehospital Adverse Traumatic Brain Injury Care Events

Adverse event	Patients, No./total No. (%)		
	Hypoxia (any prehospital Sao_2_ <90%) (n = 13 604)	Hypotension (any prehospital SBP <90 mm Hg) (n = 14 842)	Hypocarbia (any end-tidal CO_2_ <35 mm Hg) (n = 1068)
Yes, No. (%)	1577 (12)	1426 (10)	650 (61)
Hypoxia	NA	433 of 1297 (33)	189 of 607(31)
Hypotension	433 of 1557 (28)	NA	253 of 644 (39)
Hypocarbia	189 of 277 (68)	253 of 344 (74)	NA

Abbreviations: NA, not applicable; Sao_2_, arterial oxygen saturation; SBP, systolic blood pressure.

The majority (92%) of patients with TBI were not treated with advanced airway management. Of the 1264 patients (8%) treated with prehospital advanced airway management, 1133 (90%) underwent at least 1 attempt at endotracheal tube placement, 229 (18%) underwent at least 1 attempt at bag-valve mask ventilation, and 106 (8%) underwent at least 1 attempt at supraglottic airway insertion (eTable 2 in Supplement 1). At least 1 prehospital hypocarbia event was experienced by 61% of patients (650 of 1068) with advanced airway management (Table 2), and 443 (41%) were experiencing hypocarbia at the last vital signs evaluation. Among 1068 patients treated with advanced airway management, the median initial field GCS was 3 (IQR, 3-7); 888 (83%) arrived by helicopter ambulance; 671 (63%) had an ISS of at least 25, 277 (28%) had prehospital hypoxia, 344 (32%) had prehospital hypotension, and 277 (58%) had multisystem injury.

### Bivariate Associations Between Prehospital Care Event and TBI Outcomes

Death in the ED occurred in 2% (259 of 14 939) of the cohort (Table 3). Patients with hypoxia, hypotension, or hypocarbia were significantly more likely to die in the ED compared with those who did not at rates of 7% (vs 1%; *P* < .001), 8% (vs 1%; *P* < .001), and 11% (vs 1%; *P* < .001), respectively. Death in the hospital occurred in 12% of the overall study population (1764 of 14 623). Patients with hypoxia, hypotension, or hypocarbia were also significantly more likely to die in the hospital compared with those who did not, at rates of 31% (vs 9%; *P* < .001), 31% (vs 10%; *P* < .001), and 43% (vs 17%; *P* < .001), respectively (Table 3). A total of 3705 of 14 623 patients (25%) of the overall study population had an unfavorable discharge disposition (ie, death or discharge to a skilled nursing facility, hospice care, or long-term care hospital). Patients with hypoxia, hypotension, and hypocarbia had significantly higher rates of unfavorable discharge disposition compared with those who did not at 47% (vs 22%; *P* < .001), 47% (vs 23%; *P* < .001), and 56% (vs 28% [118]; *P* < .001), respectively. Among 2182 patients with a head AIS of 5 to 6, 141 (6%) died in the ER, 1032 (47%) died in the hospital, and 1351 (62%) had an unfavorable hospital discharge.

**Table 3.** Unadjusted Outcomes by Traumatic Brain Injury Adverse Prehospital Care Exposures

Outcome	No. of patients (%)									
	Overall, No./total No.	Any prehospital adverse care exposure (n = 14 994)		Hypoxia (n = 13 604)		Hypotension (n = 14 842)		Hypocarbia (n = 1068)	
		Yes (n = 2880)	No (n = 12 114)	Yes (n = 1577)	No (n = 12 017)	Yes (n = 1426)	No (n = 13 416)	Yes (n = 650)	No (n = 418)
Death in ED	259/14 939 (2)	185 (6)	74 (1)	114 (7)	113 (1)	112 (8)	140 (1)	70 (11)	6 (1)
Death in the hospital	1764/14 623 (12)	832 (29)	932 (8)	493 (31)	1099 (9)	437 (31)	1298 (10)	279 (43)	73 (17)
Unfavorable discharge disposition^a^	3705/14 623 (25)	1272 (44)	2433 (21)	734 (47)	2613 (22)	662 (47)	2992 (23)	363 (56)	118 (28)

Abbreviation: ED, emergency department.

^a^ At hospital discharge, includes death or discharge to a skilled nursing facility, hospice care, or long-term-care hospital.

### Adjusted Associations Between Prehospital Care Event and TBI Outcomes

Exposure to any prehospital TBI event was associated with an increased risk of death in the ED (adjusted RR [ARR], 2.78; 95% CI, 2.03-3.79), death in the hospital (ARR, 1.34; 95% CI, 1.25-1.45), and unfavorable discharge disposition (ARR, 1.09; 95% CI, 1.04-1.13) after accounting for known confounders (Table 4). Hypoxia (ARR, 2.24; 95% CI, 1.69-2.97), hypotension (ARR, 2.05; 95% CI, 1.54-2.72), and hypocarbia (ARR, 7.99; 95% CI, 2.47-25.85) were independently associated with increased risks of ED death. Each TBI care event was also independently associated with increased risks of hospital death and unfavorable discharge disposition. These associations were more pronounced among patients with hypocarbia or with a head AIS of 1 to 4. The relative risks were attenuated when limited to the subset of patients with a head AIS of 5 to 6 (ie, those with critical, catastrophic brain injuries) (Table 4). Patients with a combined exposure of both prehospital hypoxia and hypotension (ARR, 3.04; 95% CI, 2.11-4.39) exhibited a greater risk for death in the ED compared with those with hypoxia only (ARR, 2.24; 95% CI, 1.57-3.19) or hypotension only (ARR, 2.34; 95% CI, 1.61-3.40) (eTable 3 in Supplement 1).

**Table 4.** Associations Between Adverse Prehospital Care Exposures and Traumatic Brain Injury Outcomes, Stratified by Head AIS

Adverse prehospital care exposure	ARR^a^ (95% CI)		
	All	AIS 1-4^b^	AIS 5-6
**Death in the ED**			
Any adverse exposure	2.78 (2.03-3.79)	7.25 (4.12-12.77)	1.52 (1.05-2.21)
Hypoxia	2.24 (1.69-2.97)	5.62 (3.45-9.15)	1.31 (0.94-1.82)
Hypotension	2.05 (1.54-2.72)	3.68 (2.34-5.77)	1.33 (0.91-1.93)
Hypocarbia	7.99 (2.47-25.85)	8.70 (1.29-58.74)	7.70 (1.82-32.64)
**Death in the hospital**			
Any adverse exposure	1.34 (1.25-1.45)	1.75 (1.49-2.05)	1.24 (1.15-1.33)
Hypoxia	1.33 (1.23-1.44)	1.88 (1.60-2.19)	1.23 (1.12-1.34)
Hypotension	1.18 (1.10-1.28)	1.48 (1.28-1.71)	1.15 (1.05-1.27)
Hypocarbia	1.74 (1.35-2.25)	2.01 (1.17-3.44)	1.63 (1.24-2.15)
**Unfavorable discharge disposition**			
Any adverse exposure	1.09 (1.04-1.13)	1.28 (1.19-1.37)	1.07 (1.00-1.14)
Hypoxia	1.09 (1.04-1.14)	1.45 (1.34-1.58)	1.06 (0.98-1.15)
Hypotension	1.06 (1.02-1.10)	1.19 (1.10-1.29)	1.07 (0.99-1.15)
Hypocarbia	1.41 (1.16-1.71)	1.57 (1.09-2.27)	1.24 (1.00-1.54)

Abbreviations: AIS, Abbreviated Injury Scale (an anatomic marker for severity of traumatic brain injury); ARR, adjusted relative risk; ED, emergency department.

^a^ Relative risks were estimated using log-binomial regression models adjusted for site, sex, race and ethnicity, age, transport mode, initial field Glasgow Coma Scale score, Injury Severity Score, injury mechanism, and multiple trauma.

^b^ Due to small cells, head AIS of 1 to 2 and 3 to 4 were combined for stratification.

## Discussion

In this multicenter cohort study, prehospital hypoxia, hypotension, and hypocarbia were independently associated with higher risks of death and disability among patients with confirmed TBI, even after adjusting for demographic and injury severity confounders. These higher risks were more pronounced in patients with hypocarbia and among those with a head AIS of 1 to 4. In more severe TBI, the association of prehospital hypoxia, hypocarbia, and hypotension with death and disability was less apparent. These contemporary, multicenter, high-resolution prehospital data confirm the harmful association of hypoxia, hypotension, and hypocarbia that are the focus of the BTF prehospital TBI care guidelines.

Our study has important contrasts with the prior EPIC Study, which implemented the principles of the BTF prehospital care guidelines at more than 130 EMS agencies in Arizona, observing increased survival in patients with severe TBI, including those who underwent intubation.^9^ Ancillary studies of EPIC validated harms resulting from hypoxia, hypocarbia, and hypotension during TBI care.^9,16,17^ Our study builds on prior findings from EPIC and other studies, applying high-resolution data from a contemporary multicenter research network to verify the adverse associations of prehospital hypoxia, hypocarbia, and hypotension with TBI outcomes. Notable limitations of EPIC include its before-and-after design and the period of the dataset from 2007 to 2015, which may not reflect current prehospital practices. The direct effect of the EPIC implementation effort upon the reduction of prehospital TBI adverse events was also limited. Our analysis of current data from the LITES Network neither support nor refute the findings of EPIC. However, our observations underscore that hypoxia, hypocarbia, and hypotension are common during the prehospital care of TBI, and that their events have significant associations with poor TBI outcomes. We note that our study describes the harms of hypoxia, hypocarbia, and hypotension without accounting for efforts by EMS personnel to correct these abnormalities. Patients with a penetrating injury mechanism, a higher ISS, and/or multiple trauma TBI and who were transported by air were more likely to experience these adverse prehospital exposures, which may reflect populations with higher injury severity.

Prehospital hypoxia and hypotension combined are associated with significantly increased mortality in patients with TBI, supporting aggressive prehospital prevention and treatment of hypoxia and hypotension.^18,19^ Our findings of increased risks of death with prehospital hypotension in the context of a TBI diagnosis are consistent with several prior studies.^5,9,20,21,22^ Hypoxia in isolation has been reported to have a more nuanced, but largely negative association with outcomes in patients with TBI.^9,23,24,25^ For example, a multicenter cohort study of patients with severe TBI in Switzerland identified prehospital hypoxemia as associated with impaired consciousness (GCS ≤13) at day 14.^21^

After a patient arrives at the hospital and moderate or severe TBI is confirmed, avoidance of secondary brain injury from hypotension and hypoxia is the mainstay of treatment. At present, therapeutic options for TBI are largely supportive, aside from decompressive surgery to evacuate mass lesions and medications to reduce intracranial pressure.^6,26^ Ongoing research is needed to understand the relative impact of an isolated vs a sustained (or dose-dependent) effect of hypoxia and hypotension in patients with TBI.^5^ Early detection and correction of adverse care exposures in the prehospital setting may improve TBI outcomes in a meaningful way. Our study leverages a robust prehospital multicenter network with comprehensive repeated vital signs to underscore the importance of adherence to the BTF guidelines for prehospital TBI care.^5^ However, this study was not designed to assess the reversibility of these adverse prehospital exposures. Future clinical trials may better evaluate whether specific EMS interventions, or a combination thereof, could mitigate the impact of prehospital hypoxia, hypocarbia, and/or hypotension. These interventions may include better monitoring tools targeted to patients with a suspected TBI, the use of flow-limited bag ventilation devices, and lower thresholds for treating hypotension.

Prehospital hypocarbia is associated with deleterious outcomes in patients with severe TBI.^27^ Some debate in the literature remains on the value of prehospital intubation in patients with TBI, with meta-analyses reporting an association with higher mortality, particularly intubation at the hands of less skilled prehospital personnel.^28,29^ Most patients in our study who underwent prehospital advanced airway management also experienced hypocarbia. These patients treated with prehospital airway management also exhibited higher acuity, with a median field GCS of 3 and ISS of at least 25, and were frequently hypotensive, suggesting some selection bias. Hypocarbia can be even more deleterious in controlled environments.^30^ Nevertheless, our study shows the additive adverse association of hypocarbia, even after adjusting for confounders. Future training and quality improvement efforts should focus on avoiding hypocarbia after advanced airway management in the prehospital setting, particularly in patients suspected to have a TBI.

### Strengths and Limitations

The primary strength of our study is the robust prehospital data collection and multicenter study population, which lend broad generalizability to prehospital and emergency care of patients with suspected TBI. Our study also has several limitations. As this study was observational, we cannot determine causality, and residual or unmeasured confounding, such as differential time from injury to presentation, cannot be fully excluded. The LITES dataset is limited by both survival and spectrum bias as enrollment requires admission to the hospital and an ISS of more than 9. Patients missing prehospital vital signs were excluded out of necessity from this study, which may have introduced selection bias. Due to small cell sizes, head AIS of 1 to 2 and 3 to 4 were combined, reducing our ability to distinguish the impact of prehospital care exposures in the mild and moderate TBI categories. Just as in the original EPIC Study, it was also not possible to control for the influences of inpatient care on the outcomes of hospital mortality and unfavorable discharge disposition. We also did not evaluate adherence to the BTF bundle and, as such, were not able to show improved survival to hospital admission, improved survival to hospital discharge, or improved rates of good discharge disposition. In addition, long-term outcomes were not collected in the LITES TO1 cohort study.

## Conclusions

This cohort study found that in patients with acute TBI in the prehospital setting, hypoxia, hypocarbia, and hypotension were associated with poor outcomes. Further work is needed to establish the reversibility of these adverse prehospital care events and to better identify patients with suspected TBI for whom BTF prehospital guidelines should be applicable.

## Notes

Supplement 1. **eTable 1.** Adverse Prehospital Hypoxia and Hypotension **eTable 2.** Individuals Who Required Prehospital Advanced Airway Interventions Beyond Supplemental Oxygen **eTable 3.** Adjusted Associations Between Composite Hypoxia and Hypotension Exposure and TBI Outcomes (Relative Risk and 95% CI), Stratified by Head AIS

Supplement 2.
Data Sharing Statement
