# Supplementary material for: Adverse Prehospital Events and Outcomes After Traumatic Brain Injury
Source: JAMA Netw Open. 2025 Jan 31;8(1):e2457506. doi: 10.1001/jamanetworkopen.2024.57506 (PMC11786231; doi:10.1001/jamanetworkopen.2024.57506)
Supplement: Supplement 1. — eTable 1. Adverse Prehospital Hypoxia and Hypotension eTable 2. Individuals Who Required Prehospital Advanced Airway Interventions Beyond Supplemental Oxygen eTable 3. Adjusted Associations Between Composite Hypoxia and Hypotension Exposure and TBI Outcomes (Relative Risk and 95% CI), Stratified by Head AIS [file jamanetwopen-e2457506-s001.pdf]

Supplemental Online Content

Maiga AW, Lin H-HS, Wisniewski SR et al. Adverse prehospital events and outcomes after traumatic brain injury. *JAMA Netw Open*. 2025;8(1):e2457506. doi:10.1001/jamanetworkopen.2024.57506

eTable 1. Adverse Prehospital Hypoxia and Hypotension

eTable 2. Individuals Who Required Prehospital Advanced Airway Interventions Beyond Supplemental Oxygen

eTable 3. Adjusted Associations Between Composite Hypoxia and Hypotension Exposure and TBI Outcomes (Relative Risk and 95% CI), Stratified by Head AIS

This supplemental material has been provided by the authors to give readers additional information about their work.

© 2025 Maiga AW et al. *JAMA Network Open*.

eTable 1. Adverse Prehospital Hypoxia and Hypotension

Variables, median (25th, 75th)	**Hypoxia** (Any prehospital SaO2 <90%) n=13,604 No n=12027 (88%)	Yes n=1577 (12%)
Observation count of prehospital SaO2%	2 (1, 3)	3 (1, 6)
Minimum of prehospital SaO2%	96 (95, 99)	82 (75, 87)
	**Hypotension** (Any prehospital SBP <90 mmHg) n=14,842 No n=13416 (90%)	Yes n=1426 (10%)
Count of prehospital SBP	2 (1, 3)	4 (1, 7)
Minimum of prehospital SBP	132 (117, 150)	75 (63, 81)

© 2025 Maiga AW et al. *JAMA Network Open*.

eTable 2. Individuals who Required Prehospital Advanced Airway Interventions Beyond Supplemental Oxygen

	First intervention	Any intervention
**First advanced airway or BVM intervention**	n=14994	n=1264
No airway management interventions	13730 (92%)	
Bag Valve Mask Ventilation (BVM)	79 (1%)	229 (18%)
Endotracheal Intubation	1106 (7%)	1133 (90%)
Supraglottic Airway (SGA)	71 (0%)	106 (8%)
Surgical Airway	8 (0%)	10 (1%)

© 2025 Maiga AW et al. *JAMA Network Open*.

eTable 3. Adjusted Associations Between Composite Hypoxia and Hypotension Exposure and TBI Outcomes (Relative Risk and 95% CI), Stratified by Head AIS

Composite Exposures	Outcome	All	AIS 1-4	AIS 5-6
**Hypoxia & Hypotension**		**All**	**AIS 1-4**	**AIS 5-6**
No hypoxia or hypotension	Death in the ED	Reference	Reference	Reference
Hypoxia only		2.24 (1.57, 3.19)	5.87 (3.21, 10.73)	1.44 (0.91, 2.28)
Hypotension only		2.34 (1.61, 3.40)	5.33 (2.78, 10.25)	1.44 (0.90, 2.31)
Hypoxia and hypotension		3.04 (2.11, 4.39)	9.82 (5.26, 18.33)	1.45 (0.91, 2.33)
**Hypoxia & Hypotension**		**All**	**AIS 1-4**	**AIS 5-6**
No hypoxia or hypotension	Death in the hospital	Reference	Reference	Reference
Hypoxia only		1.35 (1.24, 1.48)	1.79 (1.48, 2.18)	1.26 (1.13, 1.40)
Hypotension only		1.23 (1.09, 1.39)	1.57 (1.29, 1.90)	1.18 (1.02, 1.36)
Hypoxia and hypotension		1.33 (1.20, 1.47)	2.36 (1.90, 2.93)	1.22 (1.08, 1.37)
**Hypoxia & Hypotension**		**All**	**AIS 1-4**	**AIS 5-6**
No hypoxia or hypotension	Unfavorable discharge disposition	Reference	Reference	Reference
Hypoxia only		1.09 (1.03, 1.14)	1.40 (1.27, 1.55)	1.05 (0.96, 1.15)
Hypotension only		1.07 (1.01, 1.13)	1.14 (1.05, 1.25)	1.13 (0.97, 1.31)
Hypoxia and hypotension		1.10 (1.03, 1.16)	1.55 (1.40, 1.72)	1.05 (0.96, 1.16)

AIS: abbreviated injury score, an anatomic marker of the severity of traumatic brain injury
Relative risk and 95%CI were estimated by log-binomial regression models adjusting for site, sex, race, age, transport mode, Initial field GCS, injury severity score, injury mechanism, and polytrauma.
Due to small cells, AIS 1-2 and 3-4 were combined for stratification.

© 2025 Maiga AW et al. *JAMA Network Open*.
