# Supplementary material for: Adverse Prehospital Events and Outcomes After Traumatic Brain Injury
Source: JAMA Netw Open. 2025 Jan 31;8(1):e2457506. doi: 10.1001/jamanetworkopen.2024.57506 (PMC11786231; doi:10.1001/jamanetworkopen.2024.57506)
Supplement: Supplement 2. — Data Sharing Statement [file jamanetwopen-e2457506-s002.pdf]

Data Sharing Statement

Maiga. Adverse Prehospital Events and Outcomes After Traumatic Brain Injury. *JAMA Netw Open*. Published January 31, 2025. doi:10.1001/jamanetworkopen.2024.57506

Data
Data available: No

Additional Information
Explanation for why data not available: Data may be shared upon reasonable request to the LITES network.
